# Case report: A 3-year follow-up study of simultaneous bilateral total hip arthroplasty for Femoral head necrosis in a patient with Kashin-Beck Disease

**DOI:** 10.3389/fsurg.2022.978697

**Published:** 2022-08-08

**Authors:** Xiangyu Chen, Haibin Liu, Houqing Wang, Liqiang Zheng, Jiayu Li, Lianqi Yan

**Affiliations:** ^1^ Medical College, Yangzhou University, Yangzhou, China; ^2^ Fu County People’s Hospital, Yan'an, China; ^3^ Dalian Medical University, Dalian, China; ^4^ Joint Department, Northern Jiangsu People’s Hospital (NJPH), Yangzhou, China

**Keywords:** Kashin-Beck Disease, femoral head necrosis, treatment, follow-up, simultaneous bilateral total hip arthroplasty

## Abstract

**Introduction:**

Kashin-Beck Disease (KBD) is an endemic disease predominantly affecting joint and skeletal muscle, predisposing the articular cartilage to degeneration and necrosis. Currently,staged total hip arthroplasty is a common surgical method for advanced femoral head necrosis from KBD, but there are no reports in the literature on simultaneous bilateral total hip arthroplasty (SB-THA) for patients with KBD.

**Case presentation:**

A 42-year-old male from Shaanxi Province, an endemic area, had bilateral hip pain for 4 years, with hips inversion and a crossed gait. After preoperative preparation, a SB-THA was performed by a posterolateral approach. Postoperative medication and functional exercises were administered and the patient was followed up for at least 3 years after discharge. The patient's hip mobility, hip scores and quality of life scores were recorded in detail during the follow-up.

**Result:**

The patient stopped antibiotic treatment on the postoperative day-2, and all inflammatory indicators showed normal and started appropriate exercise, and the pain score decreased significantly. On the postoperative day-7, the patient had gradually adapted to various forms of rehabilitation exercises. He was discharged from the hospital on the postoperative day-10 and continued to be followed up. From the preoperative period to the last follow-up, the patient's bilateral hip mobility and functional scores improved significantly, and no adverse events such as hip pain, prosthesis loosening or dislocation were found at the last follow-up.

**Conclusion:**

The patient's performance was satisfactory both intraoperatively and in the early postoperative period, but the hip scores and quality of life scores began to plateau or even decline from the third year after surgery to the last follow-up, probably due to the influence of further damage to articular cartilage in other parts of the body.

## Introduction

As a deforming osteoarthropathy with a distinct regional distribution (1), KBD was first identified and reported in eastern Siberia in 1848 (2). To date, there are about 50 suspected causative factors for KBD, but the main focus is on trace elements (selenium, iodine) deficiency theory and grain fungal toxin (T-2 toxin) poisoning theory (3, 4). KBD mainly destroys cartilage cells at the epiphysis and joints symmetrically in children, thus affecting growth and even risking deformity and disability (5). Arthroplasty is the most effective treatment for patients with advanced KBD who have failed conservative treatment and have severe joint lesions (6). It was previously believed that patients with cumulative bilateral joint lesions due to various etiologies had more complications with one-stage surgery (7).Therefore, the more symptomatic side is chosen for staged surgery. However, with the advancement in perioperative management, the improvement of orthopaedic surgeon's surgical techniques and the upgrading of orthopaedic instruments, the conditions for one-stage THA are becoming more mature and are gradually being promoted and accepted (8, 9). There is a lack of literature on simultaneous bilateral arthroplasty for KBD, but this is the first case report of SB-THA for femoral head necrosis from KBD, which is reported as follows.

## Case presentation

**Figure 1:**
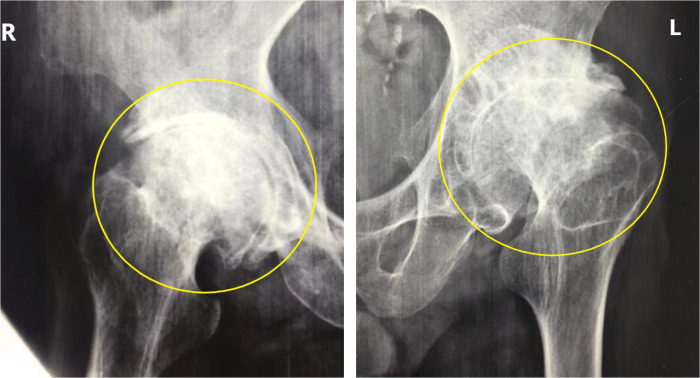
The patient's preoperative x-ray of the hip joint. (Figure notes: Circles in the x-ray were marked to show that varying degrees of necrosis of both femoral heads, with a large amount of osteophytes forming around the hip joint).

**Figure 2:**
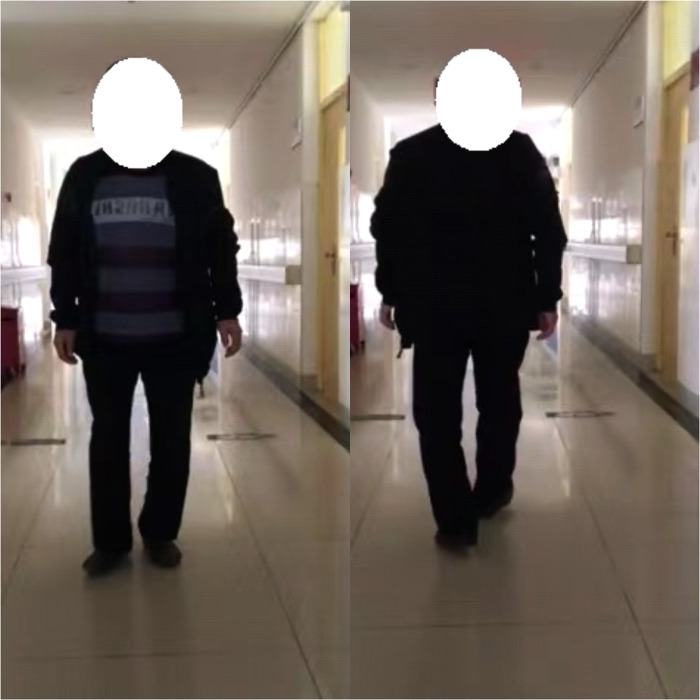
Pre-operative standing and walking status of the patient. (Figure notes: The patient had bilateral hip inversion and showed a cross gait when walking).

A 42-year-old male patient from Fu County, Shaanxi Province, an endemic area for KBD, developed bilateral hip pain about 4 years ago and was treated with pharmacological pain relief, but with poor results. In the past 1 year, the patient complained of increased pain with activity limitation, which seriously affected daily life. The x-ray examination (Figure 1) revealed bilateral aseptic necrosis of the femoral head (Ficat Type IV), and the patient had a history of KBD for more than 20 years without treatment. Specialized examination showed that both the left and right hip joints were inversion deformity with limitation of activity and pressure pain at the midpoint of the groin on both sides, and bilateral hip joint mobility was limited to different degrees in each direction (Table 1). The right and left feet were crossed (Figure 2). But blood flow and neurological reflexes were still normal in both lower limbs.

**Table 1:** Hip mobility and scores of patients before and after surgery.

Range of motion and scores	Pre-operative	1 week post-op	1 month post-op	3 months post-op	6 months post-op	1 year post-op	3 years post-op	The final follow-up
Range of motion(ROM°, Left/Right)	Flexion	45°/45°	90°/90°	120°/120°	120°/120°	125°/125°	135°/135°	135°/135°	135°/135°
	Extension	0°/0°	5°/5°	15°/15°	15°/15	15°/15	20°/20°	20°/25°	20°/25°
	Abduction	0°/0°	45°/45°	45°/50°	45°/50°	45°/50°	60°/60°	60°/60°	60°/60°
	Adduction	30°/30°	40°/40°	40°/40°	40°/40°	40°/40°	45°/45°	45°/45°	45°/45°
	内旋	10°/15°	20°/25°	20°/25	20°/25	30°/30°	30°/30°	30°/30°	30°/30°
	外旋	20°/20°	45°/45°	45°/45	45°/45	45°/45	45°/45	45°/45	45°/45°
Visual Analogue Scale		7	3	2	2	1	0	0	0
Harris score		45	80	82	87	91	98	99	97
OHS score		21	30	32	36	40	45	45	45
WOMAC score	Total	42	20	10	9	5	4	3	5
	Pain	14	5	4	4	2	0	1	2
	Stiffness	6	2	1	1	1	0	0	0
	Activity	22	13	5	4	2	2	2	3

Table Note: Harris score and OHS score are hip-specific scores, with a total score of 100 and 48, respectively. WOMAC quality of life score contains three aspects of pain, stiffness, and activity, and is divided into 24 subscales, with a total score of 96 points, from 0 to 4, in descending order of severity.

Procedure: After lumbar anesthesia, the patient was placed in the left lateral position. The surgery was performed by a posterior lateral approach (PLA). The skin, subcutaneous tissue and iliotibial bundle were incised layer by layer, the anterior edge of the gluteus maximus was incised along with the anterior edge of the gluteus maximus. The gluteus medius and gluteus minimus were incised at about 1 centimeter from the attachment of the greater trochanter. The hip joint was internally rotated and retracted. The posterior capsule of hip joint was exposed and incised with a “+” shaped incision.

The Homan pulling hook was inserted into the iliopsoas muscle stop of the lesser trochanter and pulled away. After adequate exposure of the femoral head and neck, the femoral head was found to be hypertrophied with top collapse and loss of articular cartilage.The femoral head was removed by osteotomy with a pendulum saw approximately 1.5 cm above the rotor line. After cleaning the acetabular contents, the acetabular prosthesis (52/32 mm) as well as the ceramic liner (36/44) was trial-molded and fitted. The femoral end was exposed by extreme internal rotation of the right lower extremity. After opening the marrow with a marrow opener, the femoral stalk prosthesis (2#) with ceramic ball head (36-M) was trial-molded and installed. The surgical incision was flushed and repositioned by traction and external rotation. The hip joint was moved by flexion and extension, stabilized without dislocation, and the surgical incision was closed layer by layer. Subsequently, the patient was placed in the right lateral position, and the left hip was treated in the same way. The acetabular prosthesis (52/32 mm), the ceramic liner (36/44) and the femoral stem prosthesis (1#) were installed after trial molding. Intraoperative anesthesia was satisfactory, bleeding volume was about 200 ml, no blood transfusion was given.

**Figure 3:**
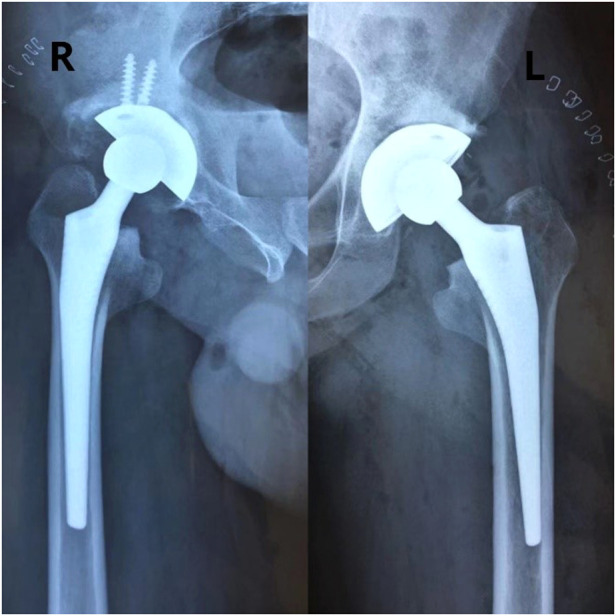
Patient's hip x-ray 1 month after surgery. (Figure notes: X-rays showed that the bilateral hip prostheses were well positioned, with no obvious dislocation or other adverse effects).

**Figure 4:**
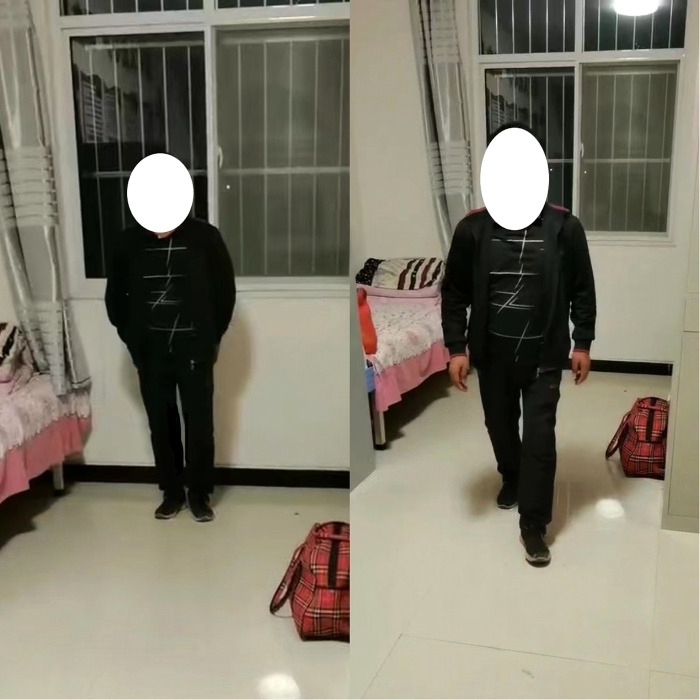
The Patient's standing and walking 1 month after surgery. (Figure notes: The patient's postoperative hip movement improved significantly and his gait was normal).

The patient stopped antibiotic treatment on the postoperative day-2, and all inflammatory indicators showed normal and started appropriate exercise, and the pain score decreased significantly. On the postoperative Day-7, the main exercises were ankle pump, hip flexion and knee flexion, and functional exercises of the hip joint with the use of assistive devices. The patient was discharged from the hospital on the postoperative day-10 and continued to be followed up. At one month after surgery, x-ray radiographs (Figure 3) revealed normal alignment and the position of the hip protheses on both sides without no signs of loosening. From the preoperative period to the last follow-up, the patient's bilateral hip mobility and functional scores improved significantly (Figure 4 and Table 1), and no adverse events such as hip pain, prosthesis loosening or dislocation were found at the final follow-up. However, the patient's hip function scores began to plateau or even decline from the third year after surgery (Harris score:99;OHS score:45), and by the last follow-up hip scores were 97 and 45 respectively. Meanwhile, the patient's WOMAC score at the final follow-up (5) was higher than in the third year after surgery (3).

## Discussion

There is no specific treatment for KBD, and when it invades joints throughout the body, the treatment protocol for osteoarthritis (OA) can be referred to. Previous literature (6) found that THA was effective in relieving pain and improving joint function after total hip arthroplasty in 22 (32 hips) elderly patients with advanced KBD. Treatment was also staged according to different symptoms and age (10). In terms of symptoms, the patient was found to be in the osteoarthritic phase (Ficat Type IV) with bilateral femoral head collapse and narrowing of the joint gap on preoperative x-ray, as well as significant clinical symptoms such as pain and impaired hip movement, which significantly limited the patient's ability to perform daily work and live. In terms of age, the young age of this patient may not meet the surgical criteria for SB-THA (11), but some scholars (12) believe that SB-THA is feasible for any patient younger than 75 years old, and considering that the symptoms of this patient are more severe than those of patients in the same age group, SB-THA may be the most effective treatment at present when conservative treatments such as braking, physiotherapy, and drugs are ineffective.

Since the first SB-THA was reported in 1971 (13), there has been a half-century-long debate about whether to choose a 1-stage THA or a staged THA. Previously, scholars (14) were concerned about the intraoperative blood loss and increased need for postoperative analgesic drugs and more complications (thromboembolic events, stroke, surgical site infection and fat embolism) associated with simultaneous surgery compared to staged surgery, However, there is now a growing consensus that SB-THA significantly reduces cumulative operative time, cumulative length of stay and total hospital costs, and also reduces adverse effects from secondary anesthetic drugs (15). In addition, compared with staged THA, SB-THA is less risky and improves the patient's hip flexion range of motion timely after surgery (16), increasing the patient's early mobility and thus improving the patient's quality of life (17). The patient in this case also started to move on the ground on the postoperative day-2, and the range of motion of the hip joint was greatly improved compared with the preoperative period (Table 1). In recent years, there has been an increasing number of different opinions regarding the comparison of complications between the two procedures. Brzezinski et al. (11) reported three cases of SB-THA with satisfactory short-term (3 months) postoperative results and no complications. The Swedish registry data (18) showed no differences in clinical outcomes and complications between one-stage THA and staged THA. Some studies (19) even concluded that SB-THA had fewer postoperative systemic complications (deep vein thrombosis, cardiovascular and pulmonary complications) and better functional outcomes (walking ability), whereas gait improvement after unilateral THA was not satisfactory (15). It is worth noting that the patient's preoperative health status has a greater impact on the overall complication rate after THA, which we generally quantified using the American Society of Anesthesiology score (ASA). Compared to ASA-1, ASA-3∼5 is one of the risk factors for increased postoperative complications (20), showing that good preoperative health is effective in avoiding postoperative comorbidities. In addition, as one of the most common complications after THA, the possibility of dislocation and loosening of components exists in every patient. An analysis of 14,314 THA cases (21) showed that the postoperative dislocation rate in patients was 1.9%, meanwhile there was no significant difference in the postoperative dislocation rate between one-stage and staged surgery (22). Whereas femoral head prosthesis diameter, acetabular component anteversion angle, and abduction angle are significantly associated with the dislocation rate of the prosthesis, 28 millimeters (mm) femoral head prosthesis is currently the most widely used, i.e., the standard head, and 32 mm or more is called large diameter femoral head, but the patient's acetabulum must be of sufficient size to be selected for fitting. When the femoral head prosthesis diameter exceeds 32 mm, it increases the jump distance and effectively reduces the dislocation rate and the need for revision (23). The dislocation rate was only 1.5% when the acetabular component had an anteversion angle of (15 ± 10)° and an abduction angle of (40 ± 10)°, while the dislocation rate beyond this safe range was 6.1% (24, 25). The diameter of the femoral head prosthesis used in our patient (Left/Right, 36 mm/36 mm), the measured postoperative acetabular component anteversion angle (Left/Right, 19.3°/20.9°), and abduction angle (Left/Right, 44.6°/44.7°) were all within these ranges, and no dislocation of the prosthesis has occurred to date. Therefore, SB-THA is increasingly recognized in terms of clinical results and complications compared with the staged THA. In addition, we should attach attention to the timely preoperative assessment of the patient's health status and the intraoperative selection of the appropriate prosthesis and optimal positioning of the acetabular component to maintain the stability of the THA.

Surgical approaches for THA include anterior approach, lateral approach (anterolateral and direct lateral) and posterior approach (posterolateral and posterior). However, there is no consensus on the selection criteria for the THA approach. PLA is traditionally used when the operator performs SB-THA, which allows extensive exposure of the surgical field, adequate osteotomy, intraoperative preservation of the gluteus medius and flexor hip groups, less damage to soft tissues, avoidance of damage to the lateral nerve vessels and reduction of nerve injury. However, intraoperative separation of the gluteus maximus muscle, which is rich in blood supply, may lead to increased intraoperative bleeding and transfusion rates (26). With the promotion of newer surgical approaches (DAA, Superpath), DAA requires only one horizontal position during one-stage surgery instead of two lateral positions by a PLA, improving the accuracy of intraoperative fluoroscopic positioning and facilitating the assessment of the position of the acetabular prosthesis and the avoidance of prosthetic dislocation and LLD (the Limb Length Discrepancy) (27, 28). Meanwhile, DAA and Superpath approach are performed through the muscle space to access the joint capsule rather than cutting or splitting the muscle, and there is no significant damage to the neuromuscular. Additionally, small surgical incision and small intraoperative bleeding are also the minimally invasive advantages of the above approaches. Although compared to PLA, DAA has been questioned due to its long learning curve and more postoperative comorbidities (29). However, as far as the overall results are concerned, the newer surgical approaches are still superior. In this case, the patient was still operated using the traditional PLA approach, probably because the patient had severe bilateral femoral head lesions with hip inversion deformity, meanwhile considering that he was a migrant worker with well-developed hip muscles due to years of labor, there would be a possibility of poor exposure of the proximal femoral surgical field and lateral femoral cutaneous nerve injury if the minimally invasive approach was adopted. Moreover, a recent randomized controlled trial (30) found no significant difference in bleeding between the two surgical approaches. Therefore, although this surgical procedure was performed by a PLA, the patient's intraoperative condition (bleeding: 200 ml; blood transfusion: none) remained satisfactory.

Although the treatment of KBD involving joint lesions follows the experience of OA, OA does not affect as many joints symmetrically as KBD, and there is less finger shortening and growth restriction (31), so the overall functional outcome of total hip replacement in patients with advanced KBD may be inferior to that of OA (6). Hip scores (Harris, OHS) also leveled off in the third year after surgery in our study patients, and quality of life scores (WOMAC) no longer increased or even tended to decrease, probably because patients undergoing SB-THA usually show similar trends in global functional outcomes over time. Instead, KBD continued to accumulate other joint lesions, which affected the patient's functional scores.

This case report follows the patient for up to 3 years, but given the patient's age, further long-term follow-up is needed to observe his postoperative outcomes and complications. We were also unable to take preoperative MRI films to further clarify the patient's diagnosis due to his personal financial level and local medical conditions. In addition, this case report is a retrospective study, which may cause some errors in the results and requires continuous improvement of the study design conditions and prospective, multicenter, and large sample studies to further elucidate.

## Conclusion

This patient with KBD was considered for SB-THA due to bilateral femoral head necrosis. Both the intraoperative specifics and the results of early postoperative rehabilitation and functional exercise were satisfactory, but from the third year to the last postoperative follow-up, hip scores and quality of life scores began to plateau or even decline, probably because large osteoarthrosis was invading other joints, causing the patient's joint lesions to worsen and thus affecting the patient's daily activities.

## Data Availability

The original contributions presented in the study are included in the article/Supplementary Material, further inquiries can be directed to the corresponding author/s.
